# Recent Advances in Disease Modeling and Drug Discovery for Diabetes Mellitus Using Induced Pluripotent Stem Cells

**DOI:** 10.3390/ijms17020256

**Published:** 2016-02-19

**Authors:** Mohammed Kawser Hossain, Ahmed Abdal Dayem, Jihae Han, Subbroto Kumar Saha, Gwang-Mo Yang, Hye Yeon Choi, Ssang-Goo Cho

**Affiliations:** Department of Animal Biotechnology, Animal Resources Research Center, and Incurable Disease Animal Model and Stem Cell Institute (IDASI), Konkuk University, Gwangjin-gu, Seoul 05029, Korea; kawsersau07@gmail.com (M.K.H.); ahmed_morsy86@yahoo.com (A.A.D.); hjh6517@naver.com (J.H.); subbroto@konkuk.ac.kr (S.K.S.); slayersgod@nate.com (G.-M.Y.); hyeon.choi24@gmail.com (H.Y.C.)

**Keywords:** diabetes mellitus, induced pluripotent stem cells, insulin-secreting β cells, cell-based drug screening, iPSC-based diabetic therapy

## Abstract

Diabetes mellitus (DM) is a widespread metabolic disease with a progressive incidence of morbidity and mortality worldwide. Despite extensive research, treatment options for diabetic patients remains limited. Although significant challenges remain, induced pluripotent stem cells (iPSCs) have the capacity to differentiate into any cell type, including insulin-secreting pancreatic β cells, highlighting its potential as a treatment option for DM. Several iPSC lines have recently been derived from both diabetic and healthy donors. Using different reprogramming techniques, iPSCs were differentiated into insulin-secreting pancreatic βcells. Furthermore, diabetes patient-derived iPSCs (DiPSCs) are increasingly being used as a platform to perform cell-based drug screening in order to develop DiPSC-based cell therapies against DM. Toxicity and teratogenicity assays based on iPSC-derived cells can also provide additional information on safety before advancing drugs to clinical trials. In this review, we summarize recent advances in the development of techniques for differentiation of iPSCs or DiPSCs into insulin-secreting pancreatic β cells, their applications in drug screening, and their role in complementing and replacing animal testing in clinical use. Advances in iPSC technologies will provide new knowledge needed to develop patient-specific iPSC-based diabetic therapies.

## 1. Introduction

Diabetes mellitus (DM), commonly referred to as diabetes, is the most widespread metabolic disease worldwide [1]. DM develops from glucose regulation defects, which cause high blood sugar levels over a prolonged period. DM leads to severe hyperglycemia and several complications, including diabetic ketoacidosis, cardiovascular disease, kidney failure, foot ulcers, and tissue or organ damage. More than three hundred million people are suffering from DM and the number of DM patients is steadily increasing.

DM is classified into three types: type 1 diabetes mellitus (T1DM), type 2 diabetes mellitus (T2DM), and gestational diabetes [2,3,4,5,6]. T1DM, previously known as insulin-dependent DM (IDDM) or juvenile diabetes, results from the destruction of insulin-secreting pancreatic β cells as a consequence of autoimmunity [2,3]. T2DM was previously known as non-insulin-dependent DM (NIDDM) or adult-onset diabetes, which is an age-related metabolic disease resulting from insulin resistance of peripheral tissues and inadequate insulin-secreting pancreatic β cell function [5,6,7]. The third form of diabetes, gestational diabetes, which occurs when pregnant women develop high blood glucose levels; however, it is usually resolved after giving birth. T1DM’s main treatment option is insulin, although administration of several classes of anti-diabetic medications, such as metformin or glucagon-like peptide-1 (GLP-1) agonists is another viable option [2,3]. Although current treatment strategies for DM are usually successful in recovering clinical status, long-term treatment could cause a variety of complications, such as cardiomyopathy, endothelial dysfunction, retinopathy, and neuropathy, which could lead to amputation and kidney diseases (resulting in renal failure) [8,9]. Replacement of insulin-secreting pancreatic β cells by islets allograft transplantation is an alternative strategy; however, this therapeutic approach is hindered by donor availability, risks of auto-immune rejection, and toxicity due to regular use of immunosuppressant drugs.

Compensation and restoration of insulin-secreting pancreatic β cell function is the most promising approach for DM. This approach can control blood glucose levels and regenerate functional insulin-secreting pancreatic β cells from adult cells [10]. However, development of this treatment has not significantly advanced owing to limited phenotypic characterization of pancreatic stem cells. Utilization of human embryonic stem cells (ESCs) is also restricted because of ethical concerns and risks of tumorigenicity [11,12,13]. Cellular reprogramming through induced pluripotent stem cell (iPSC) technology is the most advanced technology for the generation of autologous insulin-secreting pancreatic βcells. This review will summarize recent advances of pancreatic β cell differentiation from iPSCs to be used in disease modeling and drug discovery for the treatment of DM.

## 2. Generation of iPSCs

In 2006, mouse iPSCs were generated from terminally differentiated murine fibroblasts using retrovirus-mediated forced expression of the following transcription factors: Oct4 (octamer-binding transcription factor 4), Sox2 (SRY (sex determining region Y)-box 2), c-Myc (a bHLH/LZ (basic Helix-Loop-Helix Leucine Zipper) domain-containing oncogene, similar to myelocytomatosis viral oncogene (v-Myc)), and Klf4 (Kruppel-like factor 4) (OSCK or Yamanaka factors) by Shinya Yamanaka’s laboratory in Kyoto, Japan [14]. These mouse somatic fibroblast-derived iPSCs showed the genetic, epigenetic, and developmental features that were highly similar to those of mouse ESCs. In just one year, human iPSCs were successfully generated from human somatic cells by two independent research teams, J. Thomson’s [15] and S. Yamanaka’s groups [16]. Moreover, various mammalian iPSCs (human, monkeys, pigs, mice, and rats) were reported to possess the capacity for teratoma formation, which proved the pluripotency of these cells [17,18,19].

Initially, iPSC generation was carried out by a retroviral gene-delivery system using Moloney murine leukemia virus (MMLV)-derived retroviruses for stable integration of exogenous genes into the host genome [14,20,21]. A lentivirus-based reprogramming technique was also developed to generate iPSCs with higher efficiency [15,22]. In order to avoid the risk of genome instability due to the integration of retroviruses or lentiviruses, non-integrating viral vectors, such as adenoviral vectors, were used for the delivery of reprogramming transcription factors. The adenoviral vectors were allowed for a transient expression of these transcription factors [23]. However, viral vectors and other DNA-based gene delivery systems can cause critical problems in future therapeutic applications of iPSCs, including risks of insertional mutagenesis, tumorigenesis, and continued expression of potentially oncogenic proteins by integrated transgenes [20,24]. To avoid the potential risks of insertional mutagenesis in humans, generation of clinically applicable human iPSCs requires the use of technologies that can generate “footprint-free” (gene integration-free) iPSCs. To generate these footprint-free iPSCs, substantial improvements were made to non-viral delivery techniques of reprogramming factors in order to avoid integration of foreign genetic material into the host genome. Although there are still concerns about safety and efficiency, episomal non-integrating vectors [25], RNA viruses, such as the Sendai virus [26,27], continual transfection of plasmid vectors [28], lentiviruses [29] and polycistronic minicircle vectors [30] have been successfully designed to express the transcriptional factors required for the generation of footprint-free iPSCs. In addition, synthetic modified mRNAs were developed to generate RNA-iPSCs (RiPSCs) that maintained genome stability [31,32]. A nanoparticle-mediated technology to generate footprint-free iPSCs was developed using polyethylenimine (PEI)-mediated DNA [33] or RNA delivery (under revision). Treatment with various synthetic compounds (small molecules) such as valproic acid (an HDAC (histone deacetylate) inhibitor), butyrate (an HDAC inhibitor), CHIR99021 (a GSK3β (glycogen synthase kinase 3 β) inhibitor), forskolin (coleonol; an adenylyl cyclase activator), 3-deazaneplanocin A (DzNep; an *S*-adenosylmethionine-dependent methyltransferase inhibitor), tranylcypromine (a monoamine oxidase inhibitor), and TTNPB (arotinoid acid; an analog of retinoic acid (RA)) were used. A combination treatment of SB431542 (an inhibitor of ALK5 (activin receptor-like kinase 5, also known as TGFR-1 (transforming growth factor, β receptor I) or ACVRLK4 (activing receptor type II-like kinase 4)), PD0325901 (a MEK (mitogen/extracellular signal-regulated kinase or mitogen-activated protein kinase) inhibitor), and thiazovivin (a ROCK (Rho-associated coiled-coil containing protein kinase) inhibitor), was also reported. These compounds were applied to maintain or induce pluripotency, increasing cellular reprogramming efficiency or simulating the effects of iPSC-specific pluripotency genes during reprogramming [34]. Moreover, reprogramming using additional factors, such as microRNAs (miRNAs), dramatically reduced the time required to generate iPSCs and enhanced reprogramming efficiency [35]. The miRNAs are found as genome clusters and a single miRNA, which has a cell-type dependent expression pattern, can influence hundreds of mRNAs, consequently controlling cell fate. In fact, miR-302 (miRs-302a–d) and miR-367 clusters [36] or miRNAs mixtures composed of miR-302, miR-200, and miR-369 [37], were reported to significantly enhance reprogramming efficiency for iPSC generation. Therefore, iPSCs can be inexhaustible sources of cells for regenerative medicine, and have important therapeutic possibilities, which are attributable, in part, to their potential to generate pluripotent stem cells from individual diabetic patients. However, significant improvements in reprogramming techniques are required for the generation and maintenance of clinically safe and homogeneous iPSC populations.

## 3. Differentiation of iPSCs into Insulin-Secreting Pancreatic β Cells

**Figure 1:**
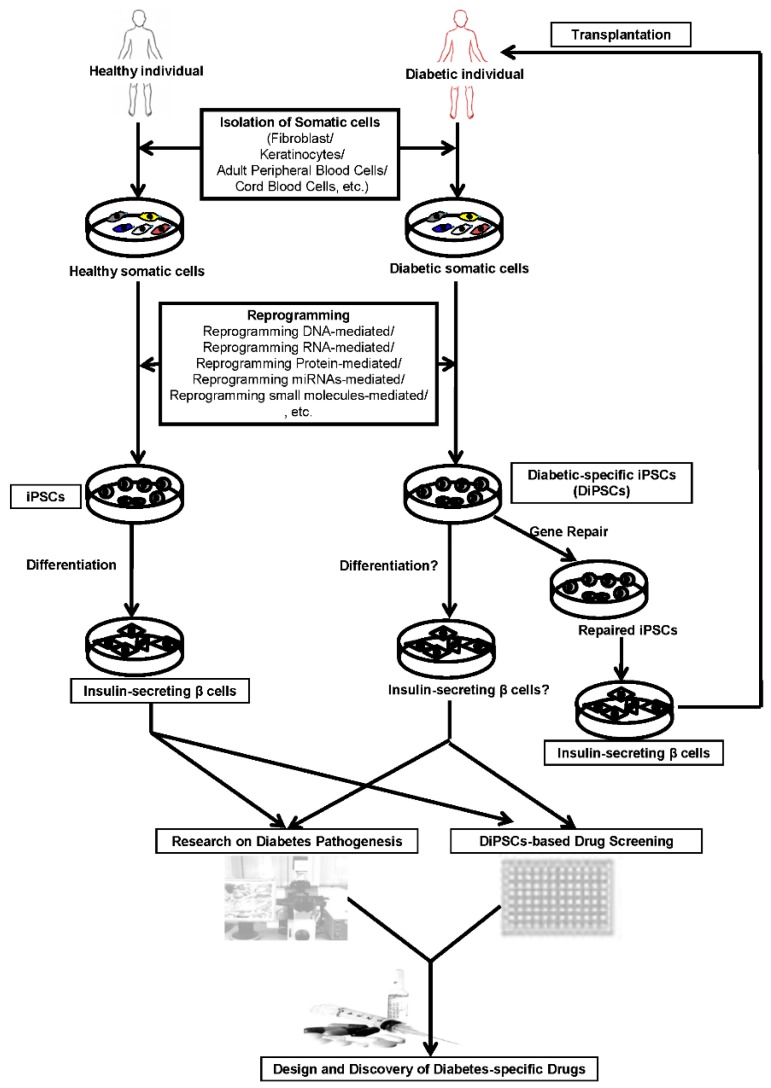
Schematic presentation of generation of iPSCs (induced pluripotent stem cells) from healthy and diabetic patients and their application in the patient-specific iPSC-based diabetic therapy. Footprint-free iPSCs can be generated from healthy individual- or diabetic patient-derived somatic cells using reprogramming DNA-, RNA-, protein-, miRNA-, or small molecule-mediated reprogramming system. DiPSCs (iPSCs derived from diabetic patients) can be further differentiated into insulin-secreting pancreatic β cells for cell-based diabetic drug screening or for transplantation into diabetic patients for cell therapy. DiPSCs can also be repaired by gene correction and then be differentiated into functional insulin-secreting pancreatic β cells, which can be transplanted into a specific diabetic patient. For disease modeling, DiPSCs can be differentiated into insulin-secreting pancreatic β cells for drug screening or pathogenesis studies to develop the compounds or therapies to treat specific type of diabetes.

Human iPSCs can be used to improve the understanding of human embryonic development and are a good source for regenerative therapies to replace damaged cells in unhealthy people. Self-renewal and differentiation capacities of human iPSCs have incited various research groups to devise novel methods and protocols for *in vitro* production of insulin-secreting pancreatic β cells [38,39,40]. Hence, human iPSCs can be generated from somatic cells of healthy individuals or diabetic patients using different iPSC generation technologies (Figure 1). Specifically, reprogramming RNA-, protein-, miRNA-, or small molecule-mediated reprogramming systems, could be used to generate clinically safe, footprint-free human iPSCs that can be differentiated into insulin-secreting pancreatic β cells. Diabetic patient-derived iPSCs (DiPSCs) can be used for cell-based diabetic drug screening or for transplantation into diabetic patients as cell therapy. Otherwise, DiPSCs can also be repaired by gene correction and differentiated into functional insulin-secreting pancreatic β cells, to be then transplanted into specific diabetic patients.

**Figure 2:**
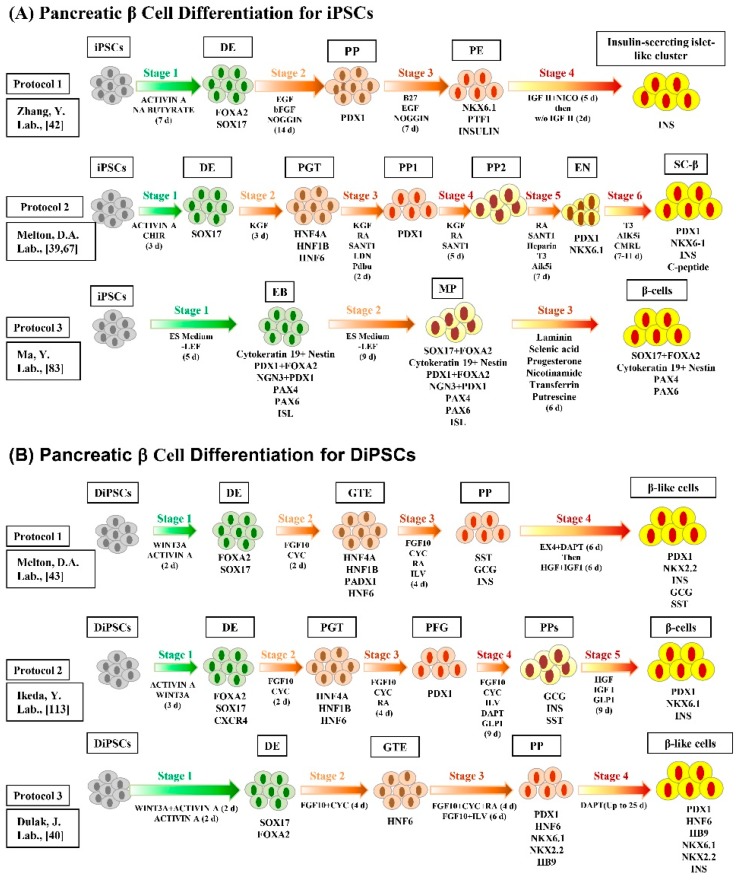
Schematic diagram depicting the various pancreatic β cell differentiation protocols for healthy iPSCs (**A**) and/or DiPSCs (**B**). The iPSCs and DiPSC can be differentiated into insulin-secreting functional β cells through the stages, embryoid body (EB), definitive endoderm (DE), pancreatic gut tube (PGT), pancreatic progenitor (PP), posterior fore gut (PFG), multi-lineage progenitor (MP), spontaneous differentiation (SD), progenitor expansion (PE), pancreatic differentiation (PD), NKX6-1^+^/C-peptide^+^ EN cells, stem cell-derived β (SC-β) cells, and/or pancreatic β-cells using specific transcription factors and small molecules. The following transcription factors, small molecules, and specific differentiation markers were used for the pancreatic β cell differentiation; EGF (epidermal growth factor), bFGF (basic fibroblast growth factor), NA butyrate (sodium butyrate), CHIR99021 (aGSK3β inhibitor), KGF (keratinocyte growth factor), RA (retinoic acid), SANT1 (a sonic hedgehog pathway antagonist), CMRL-supplement, PdBu (phorbol 12,13-dibutyrate; a PKC activator), LDN (LDN193189; a BMP pathway inhibitor), T3 (triiodothyronine; a thyroid hormone), Alk5i (ALK5 receptor inhibitor), FGF10 (fibroblast growth factor 10), CYC (cyclopamine; a Hh signaling pathway inhibitor), ILV (indolactam V; a PKC activator), SST (somatostatin; somatotropin (growth hormone) release–inhibiting hormone), GCG (glucagon), INS (insulin), HGF (hepatocyte growth factor), DAPT (a γ-secretase (NOTCH signaling pathway) inhibitor), GLP-1 (glucagon-like peptide 1), LP1 (synaptic membrane fractions), Nico (nicotinamide), FOXA2 (forkhead box protein A2), PAX4 (paired homeobox transcription factor 4), PAX 6 (paired homeobox transcription factor 6), NGN3 (neurogenin 3), HNF (hepatocyte nuclear factor), PDX1 (pancreatic and duodenal homeobox 1); NKX6.1 (NK6 homeobox transcription factor related, locus 1), SOX17 (SRY-box 17), and NKX2.2 (NK2 homeobox transcription factor related, locus 2).

Recently, various differentiation techniques were developed to generate functional insulin-secreting pancreatic β cells from iPSCs (Figure 2). These techniques involve several-week sophisticated multi-step protocol combined with several growth factors and small molecules [39,41]. These growth factors and small molecules are essential to generate mature insulin-secreting pancreatic β cells via the regulation of vital signaling pathways. In addition, a four stage serum-free *in vitro* differentiation procedure was carried out to generate insulin-secreting islet-like clusters (ILCs), which consist of C-peptide-positive and glucagon-positive cells [42]. DiPSCs were generated from the skin fibroblasts of a T1DM patient and differentiated into insulin-secreting pancreatic β cells [43]. In order to solve the problem of complication of the organogenesis process that hampers the *in vitro* derivation of organs from patient’s pluripotent stem cells, Kobayashi *et al.* succeeded to generate pluripotent stem cell-derived pancreas via compensation of the empty space of the pancreatic developmental niche by the injection of mouse wild type pluripotent stem cells into the blastocyst of the pancreatogenesis-disabled mouse (Pdx1^−/−^) [44]. Interestingly, they confirmed the possibility of interspecific chimera production between mouse and rat with injection of mouse or rat PSCs into embryos from the other species. The injected pluripotent stem cell-derived cells were distributed throughout the body and appeared to have normal function. In 2012, Ohmine *et al.* were able to generate another type of DiPSCs from the keratinocytes of an elderly T2DM patient, opening up a new venue in regenerative medicine for elder diabetic patients [45].

The DiPSCs derived from the maturity onset diabetes of the young (MODY), a monogenic form of diabetes, were also generated by Hua *et al.* in 2014 [46]. Of the 13 MODY subtypes, MODY 2 and MODY 3 are the most common forms. DiPSCs were generated from MODY2 patients, which have a mutation in the gene encoding for GCK (glucokinase). Although MODY2 patients with GCK mutations showed low glucose response sensitivity, GCK gene correction led to normal glucose sensitivity in MODY2-specific, iPSC-derived, insulin-secreting pancreatic β cells. DiPSCs from patients with different MODY subtypes (1, 2, 3, 5, and 8) were also generated [47]. MODY 1, 2, 3, 5, and 8 patients have a mutation in the gene encoding for hepatocyte nuclear factor (HNF) 4α, GCK, HNF1α, HNF1b, and bile salt dependent lipase, respectively. Additionally, high-density iPSC cultures were reported to have a crucial effect on the differentiation efficiency of pancreas-committed progenitor cells in 2015 [48].

In 2015, Stepniewski *et al.* produced DiPSCs from the murine model (lep^db/db^ (db/db) mice) or human model from patients with MODY 3 (HNF1A MODY) for evaluation and induction of the differentiation in these patient-specific iPSCs [40]. DiPSCs derived from diabetic lep^db/db^ (db/db) mice showed impaired differentiation toward the endothelial progenitor-like cells and human DiPSCs from HNF1α MODY patients failed to form teratoma [40]. Therefore, patient specific-iPSCs could be a promising tool for analysis of the disease mechanism, which are responsible for the impairment of the insulin production and vascular malfunction upon the differentiation of the DiPSCs. These findings will be helpful for future iPSC-based diabetic therapies. The efficiency of insulin-secreting pancreatic β cell differentiation has been further advanced with the generation of mature β-like cells with similar characteristics to adult β cells [49].

The differentiation of iPSCs into insulin-secreting pancreatic β cells follows a sequence of developmental stages that starts with the differentiation of definitive endoderm (DE), which is recognized by the expression of the specific endodermal markers: SOX17 (SRY-box 17), FOXA2 (forkhead box protein A2, also known as HNF3β or TCF3B (transcription factor 3B)), CXCR4 (C-X-C chemokine receptor type 4, also known as fusin or CD184 (cluster of differentiation 184)), and GSC (homeobox protein goosecoid) [49,50,51,52]. The main inducer of DE differentiation is the NODAL (nodal growth differentiation factor; a subset of the TGFβ (transforming growth factor β) superfamily) signaling pathway, which is activated by high doses of TGFβ family members such as ACTIVIN A (a member of the TGFβ family of proteins) or myostatin (also known as GDF-8 (growth differentiation factor 8)) [53,54]. The TGFβsuperfamily ligands include BMPs (bone morphogenetic proteins), GDFs (growth and differentiation factors), AMH (anti-müllerian hormone), ACTIVIN, NODAL and TGFβ's (TGFβ1, TGFβ2, and TGFβ3). TGFβ superfamily ligands bind to a type II receptor (a serine/threonine receptor kinase), which recruits and phosphorylates a type I receptor. In mammals there are seven known type I receptors and five type II receptors. The type I receptor then phosphorylates receptor-regulated SMADs (R-SMADs; SMAD1, SMAD2, SMAD3, SMAD5, and SMAD8/9) which can now bind the common-mediator SMAD (coSMAD; SMAD4). R-SMAD/coSMAD complexes accumulate in the nucleus where they act as transcription factors and participate in the regulation of target gene expression. The SMADs are homologs of the *Drosophila* proteins, mothers against decapentaplegic (MAD) and the *Caenorhabditis elegans* protein SMA (from gene *Sma* for small body size). The DE differentiation process is also stimulated by transcriptional activation of β-catenin that can be activated by wingless-type MMTV integration site family member 3A (WNT3A). Moreover, combination of ACTIVIN A with sodium butyrate (a HDAC inhibitor) or the WNT signaling activators, WNT3A or CHIR9902 (a GSK3β inhibitor), led to an increase in the efficiency of DE differentiation [50,54]. DE differentiation efficiency is also enhanced by GDF-8 or small molecules, such as IDE 1 (inducer of DE 1; an Activin/NODAL/TGF-β pathway activator) and IDE 2, which significantly induced differentiation into SOX17-expressing DE cells [55,56]. IDE1 was found to induce DE differentiation by activating the TGFβ signaling pathway. IDE1 was also reported to increase expression levels of NODAL, and induce SMAD2 phosphorylation and SOX17 expression. The DE usually generates both pancreatic and hepatic tissues in culture media. To avoid hepatic tissue differentiation, DE progenitor treatment with SU5402 (an antagonist for FGF receptor) and NOGGIN (a BMP4 antagonist, also known as NOG) directs cells to pancreatic lineage generation and pancreatic and duodenal homeobox 1, also known as insulin promoter factor 1 (PDX1) expression [57]. Retinoic acid (RA) signaling is also required for differentiation of both pancreatic and liver endoderm, which is generated from the endodermal germ layer [58,59]. DE cells can be directed to differentiate into pancreatic endocrine cells by expression of PDX1A, PTF1A (pancreas transcription factor 1 subunit α), CPA1 (carboxypeptidase A1; a monomeric pancreatic exopeptidase), SOX9 (SRY-box 9), HNF1 β, HNF4α and NKX6.1 (NK6 homeobox transcription factor related, locus 1) markers [60,61,62] and by suppression of NOTCH and Hedgehog (Hh) signaling pathways [49]. Insulin-secreting pancreatic β cells that have been generated from iPSCs were characterized by the expression panel of different factors, such as PDX1, NKX6.1, MAFA (v-maf musculoaponeurotic fibrosarcoma oncogene family, transcription factor protein A), ISL-1 (islet LIM homeodomain transcription factor; an insulin gene enhancer protein), NEUROD1 (neurogenic differentiation helix-loop-helix protein1, also known as BETA2 (β-cell E-box transactivator 2) and MODY6), GLUT2 (Glucose transporter 2, also known as solute carrier family 2 (facilitated glucose transporter), member 2 (SLC2A2)), C-peptide (a short 31-amino-acid connecting peptide between insulin’s A-chain and B-chain in the proinsulin molecule), and INS (insulin, from the *Latin*, *insula* meaning island) [50]. Specially, loss of function of NKX6.1 resulted in dysfunction in insulin synthesis and secretion, leading to diabetes as a consequence [63].Of note, over-expression of PAX4 (paired box gene 4, also known as MODY9) increased the number of insulin-secreting pancreatic β cells [64], cells that generate only one hormone (insulin), by suppressing the expression of glucagon-secreting cells [65]. The chief pancreatic regulator, PDX1, which was expressed early in embryogenesis, is downstream of forkhead box protein A1, also known as HNF3α or TCF3A (FOXA1) and FOXA2. All pancreatic cell-types were generated from PDX1-positive cells [66,67].

As previously mentioned, MODY is type of monogenic diabetes, that is distinct from the more common types of diabetes, which involve a more complex combination of causes including polygenic and environmental factors [3]. Genetic analysis demonstrated that patients suffering from a rare monogenic form of MODY showed a mutation of PDX1 and other transcription factors affecting pancreatic differentiation. Expression of the pro-endocrine bHLH transcription factor, neurogenin 3, also known as NEUROG3 (NGN3), was crucial for later stage of pancreatic development [68]. The combination of RA, cyclopamine (11-deoxojervine; an inhibitor of Hh signaling pathway), and fibroblast growth factor 10 (FGF10), was sufficient to induce pancreatic epithelium *in vitro*, from DE-derived, pluripotent stem cells by expressing PDX1 and NOGGIN, and reducing NOTCH signaling [53]. A small molecule, indolactam V (an indole alkaloid compound that activates PKC (protein kinase C)) [69] could stimulate the differentiation of PDX1-expressing pancreatic progenitor cells. Moreover, the small molecule analogs, dorsomorphin (a BMP pathway inhibitor targeting ALK2 (activin receptor-like kinase 2, also known as ACVR1 (activing A receptor, type I) or ACVRLK2), ALK3 (activin receptor-like kinase 3, also known as ACVRLK3 or BMPR1A (BMP receptor, type 1A)), and ALK6 (activin receptor-like kinase 6, also known as BMPR1B) or dorsomorphin homolog 1 (DMH1, a second-generation small molecule BMP inhibitor based on dorsomorphin), could also induce the differentiation of PDX1-expressing pancreatic progenitor cells [50,51]. Treatment with several small molecules, such as forskolin and dexamethasone [45], IGF-1 (insulin like growth factor 1), HGF (hepatocyte growth factor), and GLP-1, has been used to enhance insulin-secreting pancreatic β cells maturation *in vitro* [70].

## 4. *In Vivo* Maturation of iPSC-Derived Pancreatic β Cells

Previous studies have successfully generated, *in vitro*, insulin-secreting pancreatic β cells from either human ESCs or iPSCs [42,49,50,53,71,72,73,74,75,76,77,78]; however, differentiated cells showed a limited response to glucose. These cells lack expression of NKX6.1 and MAFA [42], specific markers of mature insulin-secreting pancreatic β cells, categorizing them as immature non-functional pancreatic β cells.

The iPSC differentiation technologies can overcome problems arising from genomic malformations, such as aberrations associated with DM, as well as problems of immune rejection [79]. Although there was a great breakthrough in the *in vitro* production of mature insulin-secreting pancreatic β cells from iPSCs, many obstacles still exist, and the capability of iPSCs to differentiate into fully functional insulin-secreting pancreatic β cells remains controversial [80,81]. Recent studies revealed that the cell’s microenvironment could play an important role for the *in vivo* generation of mature insulin-secreting pancreatic β cells [82]. Mouse embryonic and adult fibroblasts can be reprogrammed by induction of core transcription factors (Oct3/4, Sox2, c-Myc, and Klf4) to generate iPSCs, which can then be transplanted into T1DM mice model, resulting in hyperglycemia recovery [14,83]. In this regard, a number of transplantation studies have established differentiation of pancreatic progenitors into functional insulin-secreting pancreatic β cells [84]. Transplantation of iPSCs that were generated using pancreatic growth factors under kidney capsules of T1DM mice, resulted in the stabilization of serum glucose levels [85]. Moreover, transplantation of iPSCs into testicular or epididymal fat pads of immune-deficient mice pre-treated with the diabetes-inducing compound, streptozotocin (streptozocin, STZ; a glucosamine-nitrosourea compound originally discovered in a strain of the soil microbe *Streptomyces achromogenes*), led to recovery from hyperglycemia [37,46,85]. Alloxan (a toxic glucose analogue) administration in mice resulted in destruction of the pancreatic β cells and led to lack of any defense mechanisms against the chemicals that attributed to the generation superoxide free-radicals. Thus, it can be a useful tool for testing the success of transplantation of the pancreatic tissue and the cytokines-targeted therapies [86,87]. Another study showed that, when pancreatic progenitor cells were transplanted into diabetic mice within macro-encapsulation devices, they efficiently differentiated into functional mature insulin-secreting pancreatic β cells [88]. Similarly, in both T1DM and T2DM mouse models, transplanted iPSC-derived pancreatic β cells were differentiated *in vivo* into glucose-responsive pancreatic β cells that efficiently secreted insulin [83,89]. Recently, efficient differentiation procedure has been established for *in vitro* generation of hundreds of millions of β cells that were called stem cell-derived β (SC-β) cells [39]. These SC-β cells are characterized to express mature β cells markers and to showCa^2+^ flux after glucose challenge, insulin packaging, secretion of a considerable amount of insulin, and alleviation of hyperglycemia after its transplantation in diabetic mice. Therefore, the generation of SC-β cells is considered as important clinical progress that impacts the field of stem cell biology. Additionally, transplantation studies with T2DM-iPSC-derived insulin-secreting pancreatic β cells, demonstrated their clinical potential [83]. Using this approach, blood glucose levels and hyperglycemia were normalized for an extended duration. Concurrently, an increase in insulin concentration was observed *in vivo*. Interestingly, a research report proved the potency of BMP4 treatment for reprogramming gnotobiotic porcine skin-derived stem cells [4]. These cells were subsequently differentiated into insulin-producing pancreatic β cells that were able to secrete insulin after glucose stimulation. We also reported the functional activity of iPSC-derived insulin-secreting pancreatic β cells, which were reprogrammed from pancreas-derived epithelial cells of non-obese diabetic (NOD) mice, a model of autoimmune T1DM [88]. Insulin-secreting pancreatic β cells expressed several pancreatic β cell markers and secreted insulin in response to glucose stimulation. Furthermore, transplantation of iPSC-derived insulin-secreting pancreatic β cells into the kidneys of NOD mice resulted in an efficient response to glucose stimulation and a subsequent normalization of blood glucose levels. In another study, monkey iPSC-derived β cells transplanted into a diabetic mouse model led to the production of functional insulin-secreting pancreatic β cells and hyperglycemia recovery [90]. Moreover, in NOD mice, iPSC-derived insulin-secreting pancreatic β cells generated insulin in response to glucose stimulation. Other studies demonstrate that iPSC-derived pancreatic progenitor cells transplanted into mice induced the differentiation of poly hormonal cells into glucose responsive insulin-secreting pancreatic β cells [75,90,91,92]. Interestingly, it was demonstrated that the transition into mature insulin-secreting pancreatic β cells is linked to dynamic chromatin remodeling. Additionally, studies show that in young mouse islets, glucose-stimulated insulin secretion is regulated by the corticotropin-releasing factor, urocortin 3, also known as SPC (stresscopin) (Ucn3 ); a member of the sauvagine/corticotropin-releasing factor/urotensin I family), which is highly expressed in insulin-secreting pancreatic β cells [92].

## 5. Application of iPS Cell-Derived Pancreatic Cells in Diabetes Mellitus Therapy

The defect in the cellular glucose uptake in diabetic patients is attributed to the cellular inability to respond to insulin or to the lack in the insulin production. The conventional treatment of DM is based on discovery of new drugs possessing potency to lower the blood glucose level and preventing the occurrence of diabetic complications as a consequence. At the same time, the innovation of insulin is considered as an important milestone as a therapeutic tool for DM. However, in the history of medicine, the administration of insulin does not fully compensate for its loss of function. Additionally, the possibility of the failure of the clinical transplantation of pancreatic islets is ascribed to the inadequate number of donors available and the immune rejection [93]. Accordingly, the fast progression in the field of biotechnology has inspired the researchers to exert much effort on application of gene therapy, cell therapy and tissue engineering in diabetes therapy, in particular, to develop insulin producing cells using stem cell technology. In this regard, iPSC technology opens up new insight to study the differentiation and maturation of pancreatic β cells in a microenvironment similar to the body for final application in DM therapy. After the transplantation of these cells into a type 1 DM murine model, it led to decrease in the blood glucose levels in 50% of the mice [90]. This result showed the capacity of iPSCs to be transformed into insulin-producing cells, which launched the possibility for functioning of autologous transplantations in future. Transplantation of these cells results in a kidney graft with a functional response to glucose stimulation and a consequent normalization of blood glucose levels. The insulin producing β-cells transplanted under the kidney capsules of diabetic immune deficient mice gradually declined serum glucose level to either normal or near normal levels over 150 days and proved a novel option for the treatment of T1DM [85]. The transformation of DiPSCs into insulin-producing β-cells possesses various applications, such as disease mode, to study the pathogenesis of disease, for gene repair, drug screening as well as an advanced therapy [43,94,95,96]. Some drugs have already been tested on DM patient-derived iPSCs to test their therapeutic potential to alleviate disease symptoms of DM [43,94,97,98].

## 6. Disease Modeling and Drug Discovery with iPSCs

Human iPSC differentiation technologies are now considered a vital aspect to model human diseases based on patient-derived human iPSCs. Recent studies reported that different cell-types from particular disorders could be generated using patient-derived disease-specific iPSCs, which show the same disease phenotype [95,96]. In pathophysiology investigations and drug development, this breakthrough discovery has opened new venues to develop human disease models *in vitro* [94,99]. In 2015, Johannesson *et al.* reviewed that single-gene mutations in adult-differentiated cells from T1DM, T2DM, and various other types of diabetic patients, could be generated using advanced stem cell technologies [100]. These technologies can generate pluripotent cells from individuals with their own particular genetic identities, including individuals with sporadic forms of the disease [43]. These cells provide the potential to treat disorders of virtually all tissue systems in the body. Hence, disease models based on DiPSCs can be successfully applied to better understand the mechanisms of disease pathogenesis and to develop new drug treatments [43,97]. However, it would be crucial to get a representative set of human iPSCs from different patients suffering from the same disease in order to generate disease-specific DiPSC lines. It is noteworthy that patient-specific iPSC-derived insulin-secreting pancreatic β cells would still be required for disease modeling since they have the patient’s immune markers, to avoid potential immunological rejection of the cells. In addition, in patient-specific DiPSCs, disease-causing gene mutations can also be repaired by gene targeting [98]. Thus, these repaired cells can be differentiated into targeted cells, which can then be used for transplantation into the patient and alleviate disease symptoms. It may be also important to refer to the role of the mitochondria in the pathogenesis of the diabetes. The mitochondrial DNA (mtDNA) mutation, A3243G, is considered the most common mtDNA mutation that related to the occurrence of various diseases including DM [29,101] and a research group successfully generated iPSC from patients harboring mtDNA A3243G mutation [102]. Accordingly, these interesting research trials can afford a promising model for anti-diabetic drug screening and autologous transplantation therapy.

Before using novel drugs as treatments, their potential toxic effects need to be evaluated with laboratory animals, such as mice, dogs, and pigs [97,98]. These tests are very expensive, not efficiently standardized, and significantly different effects between humans and animals can be obtained. Therefore, patient-specific DiPSCs could be a novel disease model to test the effectiveness of DM drug treatments. These advances will significantly facilitate pharmacology and toxicology research of novel drugs developed as DM treatment [43,88,89]. In fact, some drugs have already been tested on DM patient-derived iPSCs. These novel drugs show therapeutic potential by alleviating disease symptoms in DM patient-specific iPSCs. To use iPSC technologies, it would have to be possible to generate DiPSCs from T1DM individuals, while studying the mechanisms regarding autoimmune disease by preparing differentiated cell-types involved in pathogenesis including: thymus epithelial cells, dendritic cells, various types of T-cells, and especially insulin-secreting pancreatic β cells. Like T1DM, T2DM derived-iPSCs could be of use in studies of insulin-secreting pancreatic β cells owing to their genetic associations with diseases and pharmacology.

## 7. Current Challenges and Future Perspectives

DM is one disorder that could potentially be treated using stem cell therapy. Specifically, iPSC technologies have provided a remarkable breakthrough to generate functional insulin-secreting pancreatic β cells, as well as patient-specific DiPSCs (Table 1). However, there are a lot of gaps and challenges that have to be addressed to be able to use iPSC-based diabetic therapies. One of the main limitations in the cell-based drug screening technology is the lack of supply of the pancreatic islets. Developing a simple, reproducible, and effective method is the greatest challenge for iPSC production [97,98]. To date, several studies have been performed to improve differentiation techniques that can generate glucose-responsive insulin-secreting pancreatic β cells to treat DM; however, glucose responses are very limited owing to their immature nature [50,51]. Thus, continuous research is required to develop differentiation protocols that exclusively generate mature insulin-secreting pancreatic β cells, which are necessary for disease modeling and cell therapy, and, in the future, to be used as a therapeutic option.

Genomic stability of iPSCs needs to be maintained, as mutations could occur by the use of harmful genome-integrating viruses, which are associated with reprogramming processes [103,104,105,106]. These processes could suppress or delete some genes in order to enhance reprogramming efficiency, negatively influencing diabetic patient iPSCs research. Therefore, differentiation techniques to develop insulin-secreting pancreatic β cells should be highly specific, competent, and should not leave any undifferentiated cells, since it can result in teratoma formation.

Another limitation of iPSC-based diabetic therapies is the low production efficiency of insulin-secreting pancreatic β cells. Hence, future research should be carried out to elucidate molecular mechanisms that lead to effective differentiation protocols, resulting in the production of high-density cultures of insulin-secreting pancreatic β cells. Moreover, another challenge in iPSCs studies is undesired cell growth. Recent studies suggested that encapsulation of the transplanted cells can prevent overgrowth *in vivo* [107,108]. Additionally, encapsulation could avoid contamination with undifferentiated cells after iPSCs differentiation into insulin-secreting pancreatic β cells [90,109]. Furthermore, in each stage of insulin-secreting pancreatic β cell differentiation, genome-wide transcriptional analysis should be done to identify defects in transcription factors. It is also important to recognize differences in gene expression, comparing *in vivo* pancreatic development with *in vitro* pancreatic differentiation [47,48,110,111,112,113]. Therefore, signaling pathways controlling the differentiation process *in vitro* should be used to develop diverse differentiation protocols.

## 8. Conclusions

In DM, insulin-secreting pancreatic β cell restoration can be achieved by two novel strategies, cell regeneration and cell replacement; the latter involves the transplantation of iPSC-derived insulin-secreting pancreatic β cells. Recent advances in stem cell therapy using iPSCs have opened new research venues for DM therapies. However, drug safety is a significant concern with these cell-types, which could delay their clinical application; thus, both *in vitro* and *in vivo* evaluations are necessary to understand their potential as diabetic therapies. Despite these challenges and obstacles, additional research efforts and progress in DiPSC-based diabetic drug discovery and iPSC-derived insulin-secreting pancreatic β cell-mediated therapies are needed to make iPSC-based therapeutic approaches feasible for DM treatment in the near future.

## Figures and Tables

**Table 1:** The application of iPSCs and DiPSCs for DM therapy or DiPSC-based diabetic drug screening.

	iPSCs	References	DiPSCs	References
**Application**	Applied in cell based therapy in diabetes mellitus	[42]	Disease modeling of diabetes mellitus	[43]
	Transplantation in diabetic patient	[88,90]	Pathogenesis of disease genotype and phenotype	[43]
			Drug screening for treatment of diabetes mellitus	[42,43]
			Autologous cell replacement therapies in case of T2DM	[43,45]
**Positive Points**	Can overcome immune rejection	[14,15,16,17,18,19]	Overcome barrier of immune rejection	[42,43,45]
	Clinically safe	[14,15,16,42]	Identify genome aberration	[103,104]
	Ideal source for transplantation therapy	[111]	Gradually engrafted in transplantation	[83,88,89]
	Stably engrafted in transplantation	[88,89]	Secrete insulin upon glucose stimulation	[83]
	Secrete insulin according to the physiological and pathological condition	[83]		
**Negative Points**	Generate complex cell population	[114]	Relatively low differentiation efficiency and high cost	[114]
	Lack of monitoring the safety and the long term efficacy	[115]	Immature phenotypes of derived islets	[38]
	Lack of understanding the signaling pathways that direct β cell maturation *in vivo*	[82,114]	Deficiency in monitoring the safety and the long term efficacy	[115]
	Teratoma formation	[38]	Sustained autoimmunity in T1D (not in T2D) can reject iPSC-derived islets	[114,116]

## References

[1] Abdulazeez S.S. (2013). Diabetes treatment: A rapid review of the current and future scope of stem cell research. Saudi Pharm. J..

[2] Atkinson M.A., Eisenbarth G.S. (2001). Type 1 diabetes: New perspectives on disease pathogenesis and treatment. Lancet.

[3] Ashcroft F.M., Rorsman P. (2012). Diabetes mellitus and the β cell: The last ten years. Cell.

[4] Yang J.H., Lee S.H., Heo Y.T., Uhm S.J., Lee H.T. (2010). Generation of insulin-producing cells from gnotobiotic porcine skin-derived stem cells. Biochem. Biophys. Res. Commun..

[5] Defronzo R.A. (1997). Pathogenesis of type 2 diabetes: Metabolic and molecular implications for identifying diabetes genes. Diabetes Rev..

[6] Paneni F., Beckman J.A., Creager M.A., Cosentino F. (2013). Diabetes and vascular disease: Pathophysiology, clinical consequences, and medical therapy: Part i. Eur. Heart J..

[7] Choi S.B., Lee J.H., Kim S., Han S.D., Kim I.H., Noh Y.H. (2013). Improvement of β-cell function after achievement of optimal glycaemic control via long-term continuous subcutaneous insulin infusion therapy in non-newly diagnosed type 2 diabetic patients with suboptimal glycaemic control. Diabetes Metab. Res. Rev..

[8] Ali M.A., Dayan C.M. (2009). Review: The importance of residual endogenous β-cell preservation in type 1 diabetes. Br. J. Diabetes Vasc. Dis..

[9] Powers A.C., D'alessio D. (2011). Endocrine Pancreas and Pharmacotherapy of Diabetes Mellitus and Hypoglycemia.

[10] Vetere A., Choudhary A., Burns S.M., Wagner B.K. (2014). Targeting the pancreatic β-cell to treat diabetes. Nat. Rev. Drug Discov..

[11] Soria B., Roche E., Berna G., Leon-Quinto T., Reig J.A., Martin F. (2000). Insulin-secreting cells derived from embryonic stem cells normalize glycemia in streptozotocin-induced diabetic mice. Diabetes.

[12] Assady S., Maor G., Amit M., Itskovitz-Eldor J., Skorecki K.L., Tzukerman M. (2001). Insulin production by human embryonic stem cells. Diabetes.

[13] Yao S., Chen S., Clark J., Hao E., Beattie G.M., Hayek A., Ding S. (2006). Long-term self-renewal and directed differentiation of human embryonic stem cells in chemically defined conditions. Proc. Natl. Acad. Sci. USA.

[14] Takahashi K., Yamanaka S. (2006). Induction of pluripotent stem cells from mouse embryonic and adult fibroblast cultures by defined factors. Cell.

[15] Yu J., Vodyanik M.A., Smuga-Otto K., Antosiewicz-Bourget J., Frane J.L., Tian S., Nie J., Jonsdottir G.A., Ruotti V., Stewart R. (2007). Induced pluripotent stem cell lines derived from human somatic cells. Science.

[16] Takahashi K., Tanabe K., Ohnuki M., Narita M., Ichisaka T., Tomoda K., Yamanaka S. (2007). Induction of pluripotent stem cells from adult human fibroblasts by defined factors. Cell.

[17] Ezashi T., Telugu B.P., Alexenko A.P., Sachdev S., Sinha S., Roberts R.M. (2009). Derivation of induced pluripotent stem cells from pig somatic cells. Proc. Natl. Acad. Sci. USA.

[18] Li W., Wei W., Zhu S., Zhu J., Shi Y., Lin T., Hao E., Hayek A., Deng H., Ding S. (2009). Generation of rat and human induced pluripotent stem cells by combining genetic reprogramming and chemical inhibitors. Cell Stem Cell.

[19] Liao J., Cui C., Chen S., Ren J., Chen J., Gao Y., Li H., Jia N., Cheng L., Xiao H. (2009). Generation of induced pluripotent stem cell lines from adult rat cells. Cell Stem Cell.

[20] Okita K., Ichisaka T., Yamanaka S. (2007). Generation of germline-competent induced pluripotent stem cells. Nature.

[21] Stadtfeld M., Maherali N., Borkent M., Hochedlinger K. (2010). A reprogrammable mouse strain from gene-targeted embryonic stem cells. Nat. Methods.

[22] Blelloch R., Venere M., Yen J., Ramalho-Santos M. (2007). Generation of induced pluripotent stem cells in the absence of drug selection. Cell Stem Cell.

[23] Stadtfeld M., Nagaya M., Utikal J., Weir G., Hochedlinger K. (2008). Induced pluripotent stem cells generated without viral integration. Science.

[24] Sun N., Longaker M.T., Wu J.C. (2010). Human iPS cell-based therapy: Considerations before clinical applications. Cell Cycle.

[25] Yu J., Hu K., Smuga-Otto K., Tian S., Stewart R., Slukvin I.I., Thomson J.A. (2009). Human induced pluripotent stem cells free of vector and transgene sequences. Science.

[26] Fusaki N., Ban H., Nishiyama A., Saeki K., Hasegawa M. (2009). Efficient induction of transgene-free human pluripotent stem cells using a vector based on sendai virus, an RNA virus that does not integrate into the host genome. Proc. Jpn. Acad. Ser. B-Phys. Biol. Sci..

[27] Seki T., Yuasa S., Fukuda K. (2012). Generation of induced pluripotent stem cells from a small amount of human peripheral blood using a combination of activated T cells and Sendai virus. Nat. Protoc..

[28] Okita K., Hong H., Takahashi K., Yamanaka S. (2010). Generation of mouse-induced pluripotent stem cells with plasmid vectors. Nat. Protoc..

[29] Kodaira M., Hatakeyama H., Yuasa S., Seki T., Egashira T., Tohyama S., Kuroda Y., Tanaka A., Okata S., Hashimoto H. (2015). Impaired respiratory function in melas-induced pluripotent stem cells with high heteroplasmy levels. FEBS Open Bio.

[30] Jia F., Wilson K.D., Sun N., Gupta D.M., Huang M., Li Z., Panetta N.J., Chen Z.Y., Robbins R.C., Kay M.A. (2010). A nonviral minicircle vector for deriving human iPS cells. Nat. Methods.

[31] Warren L., Manos P.D., Ahfeldt T., Loh Y.H., Li H., Lau F., Ebina W., Mandal P.K., Smith Z.D., Meissner A. (2010). Highly efficient reprogramming to pluripotency and directed differentiation of human cells with synthetic modified mRNA. Cell Stem Cell.

[32] Walia B., Satija N., Tripathi R.P., Gangenahalli G.U. (2012). Induced pluripotent stem cells: Fundamentals and applications of the reprogramming process and its ramifications on regenerative medicine. Stem Cell Rev. Rep..

[33] Lee C.H., Kim J.H., Lee H.J., Jeon K., Lim H., Choi H., Lee E.R., Park S.H., Park J.Y., Hong S. (2011). The generation of iPS cells using non-viral magnetic nanoparticle based transfection. Biomaterials.

[34] Lin T., Ambasudhan R., Yuan X., Li W., Hilcove S., Abujarour R., Lin X., Hahm H.S., Hao E., Hayek A. (2009). A chemical platform for improved induction of human iPSCs. Nat. Methods.

[35] Sridharan R., Plath K. (2011). Small RNAs loom large during reprogramming. Cell Stem Cell.

[36] Anokye-Danso F., Trivedi C.M., Juhr D., Gupta M., Cui Z., Tian Y., Zhang Y., Yang W., Gruber P.J., Epstein J.A. (2011). Highly efficient miRNA-mediated reprogramming of mouse and human somatic cells to pluripotency. Cell Stem Cell.

[37] Miyoshi N., Ishii H., Nagano H., Haraguchi N., Dewi D.L., Kano Y., Nishikawa S., Tanemura M., Mimori K., Tanaka F. (2011). Reprogramming of mouse and human cells to pluripotency using mature microRNAs. Cell Stem Cell.

[38] Soejitno A., Prayudi P.K. (2011). The prospect of induced pluripotent stem cells for diabetes mellitus treatment. Ther. Adv. Endocrinol. Metab..

[39] Pagliuca F.W., Millman J.R., Gurtler M., Segel M., van Dervort A., Ryu J.H., Peterson Q.P., Greiner D., Melton D.A. (2014). Generation of functional human pancreatic β cells *in vitro*. Cell.

[40] Stepniewski J., Kachamakova-Trojanowska N., Ogrocki D., Szopa M., Matlok M., Beilharz M., Dyduch G., Malecki M.T., Jozkowicz A., Dulak J. (2015). Induced pluripotent stem cells as a model for diabetes investigation. Sci. Rep..

[41] Rezania A., Bruin J.E., Arora P., Rubin A., Batushansky I., Asadi A., O'Dwyer S., Quiskamp N., Mojibian M., Albrecht T. (2014). Reversal of diabetes with insulin-producing cells derived *in vitro* from human pluripotent stem cells. Nat. Biotechnol..

[42] Tateishi K., He J., Taranova O., Liang G., D'Alessio A.C., Zhang Y. (2008). Generation of insulin-secreting islet-like clusters from human skin fibroblasts. J. Biol. Chem..

[43] Maehr R., Chen S., Snitow M., Ludwig T., Yagasaki L., Goland R., Leibel R.L., Melton D.A. (2009). Generation of pluripotent stem cells from patients with type 1 diabetes. Proc. Natl. Acad. Sci. USA.

[44] Kobayashi T., Yamaguchi T., Hamanaka S., Kato-Itoh M., Yamazaki Y., Ibata M., Sato H., Lee Y.S., Usui J., Knisely A.S. (2010). Generation of rat pancreas in mouse by interspecific blastocyst injection of pluripotent stem cells. Cell.

[45] Ohmine S., Squillace K.A., Hartjes K.A., Deeds M.C., Armstrong A.S., Thatava T., Sakuma T., Terzic A., Kudva Y., Ikeda Y. (2012). Reprogrammed keratinocytes from elderly type 2 diabetes patients suppress senescence genes to acquire induced pluripotency. Aging-US.

[46] Hua X.F., Wang Y.W., Tang Y.X., Yu S.Q., Jin S.H., Meng X.M., Li H.F., Liu F.J., Sun Q., Wang H.Y. (2014). Pancreatic insulin-producing cells differentiated from human embryonic stem cells correct hyperglycemia in scid/nod mice, an animal model of diabetes. PLoS ONE.

[47] Teo A.K., Windmueller R., Johansson B.B., Dirice E., Njolstad P.R., Tjora E., Raeder H., Kulkarni R.N. (2013). Derivation of human induced pluripotent stem cells from patients with maturity onset diabetes of the young. J. Biol. Chem..

[48] Toyoda T., Mae S., Tanaka H., Kondo Y., Funato M., Hosokawa Y., Sudo T., Kawaguchi Y., Osafune K. (2015). Cell aggregation optimizes the differentiation of human ESCs and iPSCs into pancreatic bud-like progenitor cells. Stem Cell Res..

[49] Jiang J., Au M., Lu K., Eshpeter A., Korbutt G., Fisk G., Majumdar A.S. (2007). Generation of insulin-producing islet-like clusters from human embryonic stem cells. Stem Cells.

[50] Zhang D., Jiang W., Liu M., Sui X., Yin X., Chen S., Shi Y., Deng H. (2009). Highly efficient differentiation of human ES cells and iPS cells into mature pancreatic insulin-producing cells. Cell Res..

[51] Chen S., Borowiak M., Fox J.L., Maehr R., Osafune K., Davidow L., Lam K., Peng L.F., Schreiber S.L., Rubin L.L. (2009). A small molecule that directs differentiation of human ESCs into the pancreatic lineage. Nat. Chem. Biol..

[52] Jennings R.E., Berry A.A., Kirkwood-Wilson R., Roberts N.A., Hearn T., Salisbury R.J., Blaylock J., Piper Hanley K., Hanley N.A. (2013). Development of the human pancreas from foregut to endocrine commitment. Diabetes.

[53] D'Amour K.A., Bang A.G., Eliazer S., Kelly O.G., Agulnick A.D., Smart N.G., Moorman M.A., Kroon E., Carpenter M.K., Baetge E.E. (2006). Production of pancreatic hormone-expressing endocrine cells from human embryonic stem cells. Nat. Biotechnol..

[54] McLean A.B., D'Amour K.A., Jones K.L., Krishnamoorthy M., Kulik M.J., Reynolds D.M., Sheppard A.M., Liu H., Xu Y., Baetge E.E. (2007). Activin a efficiently specifies definitive endoderm from human embryonic stem cells only when phosphatidylinositol 3-kinase signaling is suppressed. Stem Cells.

[55] Borowiak M., Maehr R., Chen S., Chen A.E., Tang W., Fox J.L., Schreiber S.L., Melton D.A. (2009). Small molecules efficiently direct endodermal differentiation of mouse and human embryonic stem cells. Cell Stem Cell.

[56] Hosoya M. (2012). Preparation of pancreatic β-cells from human iPS cells with small molecules. Islets.

[57] Wandzioch E., Zaret K.S. (2009). Dynamic signaling network for the specification of embryonic pancreas and liver progenitors. Science.

[58] Martin M., Gallego-Llamas J., Ribes V., Kedinger M., Niederreither K., Chambon P., Dolle P., Gradwohl G. (2005). Dorsal pancreas agenesis in retinoic acid-deficient raldh2 mutant mice. Dev. Biol..

[59] Molotkov A., Molotkova N., Duester G. (2005). Retinoic acid generated by raldh2 in mesoderm is required for mouse dorsal endodermal pancreas development. Dev. Dyn..

[60] Kopp J.L., Dubois C.L., Schaffer A.E., Hao E., Shih H.P., Seymour P.A., Ma J., Sander M. (2011). Sox9+ ductal cells are multipotent progenitors throughout development but do not produce new endocrine cells in the normal or injured adult pancreas. Development.

[61] Schaffer A.E., Freude K.K., Nelson S.B., Sander M. (2010). Nkx6 transcription factors and Ptf1a function as antagonistic lineage determinants in multipotent pancreatic progenitors. Dev. Cell.

[62] Rezania A., Bruin J.E., Xu J., Narayan K., Fox J.K., O'Neil J.J., Kieffer T.J. (2013). Enrichment of human embryonic stem cell-derived Nkx6.1-expressing pancreatic progenitor cells accelerates the maturation of insulin-secreting cells *in vivo*. Stem Cells.

[63] Taylor B.L., Liu F.F., Sander M. (2013). Nkx6.1 is essential for maintaining the functional state of pancreatic β cells. Cell Rep..

[64] Gage B.K., Baker R.K., Kieffer T.J. (2014). Overexpression of PAX4 reduces glucagon expression in differentiating hESCs. Islets.

[65] Kunisada Y., Tsubooka-Yamazoe N., Shoji M., Hosoya M. (2012). Small molecules induce efficient differentiation into insulin-producing cells from human induced pluripotent stem cells. Stem Cell Res..

[66] Gannon M., Herrera P.L., Wright C.V. (2000). Mosaic Cre-mediated recombination in pancreas using the *pdx-1* enhancer/promoter. Genesis.

[67] Gu G., Dubauskaite J., Melton D.A. (2002). Direct evidence for the pancreatic lineage: Ngn3+ cells are islet progenitors and are distinct from duct progenitors. Development.

[68] Zhao Y. (2015). Developing with functional β cells to treat diabetes. Int. J. Transl. Sci..

[69] Thatava T., Nelson T.J., Edukulla R., Sakuma T., Ohmine S., Tonne J.M., Yamada S., Kudva Y., Terzic A., Ikeda Y. (2011). Indolactam V/GLP-1-mediated differentiation of human iPS cells into glucose-responsive insulin-secreting progeny. Gene Ther..

[70] Kroon E., Martinson L.A., Kadoya K., Bang A.G., Kelly O.G., Eliazer S., Young H., Richardson M., Smart N.G., Cunningham J. (2008). Pancreatic endoderm derived from human embryonic stem cells generates glucose-responsive insulin-secreting cells *in vivo*. Nat. Biotechnol..

[71] Nostro M.C., Sarangi F., Ogawa S., Holtzinger A., Corneo B., Li X., Micallef S.J., Park I.-H., Basford C., Wheeler M.B. (2011). Stage-specific signaling through TGFΒ family members and Wnt regulates patterning and pancreatic specification of human pluripotent stem cells. Development.

[72] Shim J.H., Kim S.E., Woo D.H., Kim S.K., Oh C.H., McKay R., Kim J.H. (2007). Directed differentiation of human embryonic stem cells towards a pancreatic cell fate. Diabetologia.

[73] Mfopou J.K., Chen B., Mateizel I., Sermon K., Bouwens L. (2010). Noggin, retinoids, and fibroblast growth factor regulate hepatic or pancreatic fate of human embryonic stem cells. Gastroenterology.

[74] Kelly O.G., Chan M.Y., Martinson L.A., Kadoya K., Ostertag T.M., Ross K.G., Richardson M., Carpenter M.K., D'Amour K.A., Kroon E. (2011). Cell-surface markers for the isolation of pancreatic cell types derived from human embryonic stem cells. Nat. Biotechnol..

[75] Cai J., Yu C., Liu Y., Chen S., Guo Y., Yong J., Lu W., Ding M., Deng H. (2010). Generation of homogeneous PDX1(+) pancreatic progenitors from human ES cell-derived endoderm cells. J. Mol. Cell. Biol..

[76] Bruin J.E., Rezania A., Xu J., Narayan K., Fox J.K., O'Neil J.J., Kieffer T.J. (2013). Maturation and function of human embryonic stem cell-derived pancreatic progenitors in macroencapsulation devices following transplant into mice. Diabetologia.

[77] Pellegrini S., Ungaro F., Mercalli A., Melzi R., Sebastiani G., Dotta F., Broccoli V., Piemonti L., Sordi V. (2015). Human induced pluripotent stem cells differentiate into insulin-producing cells able to engraft *in vivo*. Acta Diabetol..

[78] Shaer A., Azarpira N., Vahdati A., Karimi M.H., Shariati M. (2015). Differentiation of human-induced pluripotent stem cells into insulin-producing clusters. Exp. Clin. Transplant..

[79] Pandian G.N., Taniguchi J., Sugiyama H. (2014). Cellular reprogramming for pancreatic β-cell regeneration: Clinical potential of small molecule control. Clin. Transl. Med..

[80] Brolen G.K., Heins N., Edsbagge J., Semb H. (2005). Signals from the embryonic mouse pancreas induce differentiation of human embryonic stem cells into insulin-producing β-cell-like cells. Diabetes.

[81] Segev H., Fishman B., Ziskind A., Shulman M., Itskovitz-Eldor J. (2004). Differentiation of human embryonic stem cells into insulin-producing clusters. Stem Cells.

[82] Abdelalim E.M., Emara M.M. (2015). Advances and challenges in the differentiation of pluripotent stem cells into pancreatic β cells. World J. Stem Cells.

[83] Alipio Z., Liao W., Roemer E.J., Waner M., Fink L.M., Ward D.C., Ma Y. (2010). Reversal of hyperglycemia in diabetic mouse models using induced-pluripotent stem (iPS)-derived pancreatic β-like cells. Proc. Natl. Acad. Sci. USA.

[84] Russ H.A., Parent A.V., Ringler J.J., Hennings T.G., Nair G.G., Shveygert M., Guo T., Puri S., Haataja L., Cirulli V. (2015). Controlled induction of human pancreatic progenitors produces functional β-like cells *in vitro*. Embo J..

[85] Raikwar S.P., Kim E.M., Sivitz W.I., Allamargot C., Thedens D.R., Zavazava N. (2015). Human iPS cell-derived insulin producing cells form vascularized organoids under the kidney capsules of diabetic mice. PLoS ONE.

[86] Gerace D., Martiniello-Wilks R., Simpson A., Vinuthinee N., Azreen-Redzal A., Juanarita J., Zunaina E., Lam J., Wong T., Tong L. (2015). Diabetes reversal via gene transfer: Building on successes in animal models. Res. Rep. Endocr. Disord..

[87] Szkudelski T. (2001). The mechanism of alloxan and streptozotocin action in b cells of the rat pancreas. Physiol. Res..

[88] Jeon K., Lim H., Kim J.H., Thuan N.V., Park S.H., Lim Y.M., Choi H.Y., Lee E.R., Lee M.S., Cho S.G. (2012). Differentiation and transplantation of functional pancreatic β cells generated from induced pluripotent stem cells derived from a type 1 diabetes mouse model. Stem Cells Dev..

[89] Rezania A., Riedel M.J., Wideman R.D., Karanu F., Ao Z., Warnock G.L., Kieffer T.J. (2011). Production of functional glucagon-secreting α-cells from human embryonic stem cells. Diabetes.

[90] Zhu F.F., Zhang P.B., Zhang D.H., Sui X., Yin M., Xiang T.T., Shi Y., Ding M.X., Deng H. (2011). Generation of pancreatic insulin-producing cells from rhesus monkey induced pluripotent stem cells. Diabetologia.

[91] Xie R., Everett L.J., Lim H.W., Patel N.A., Schug J., Kroon E., Kelly O.G., Wang A., D'Amour K.A., Robins A.J. (2013). Dynamic chromatin remodeling mediated by polycomb proteins orchestrates pancreatic differentiation of human embryonic stem cells. Cell Stem Cell.

[92] Li C., Chen P., Vaughan J., Lee K.F., Vale W. (2007). Urocortin 3 regulates glucose-stimulated insulin secretion and energy homeostasis. Proc. Natl. Acad. Sci. USA.

[93] Azzi J., Geara A.S., El-Sayegh S., Abdi R. (2010). Immunological aspects of pancreatic islet cell transplantation. Expert Rev. Clin. Immunol..

[94] Rashid S.T., Corbineau S., Hannan N., Marciniak S.J., Miranda E., Alexander G., Huang-Doran I., Griffin J., Ahrlund-Richter L., Skepper J. (2010). Modeling inherited metabolic disorders of the liver using human induced pluripotent stem cells. J. Clin. Investig..

[95] Itzhaki I., Maizels L., Huber I., Zwi-Dantsis L., Caspi O., Winterstern A., Feldman O., Gepstein A., Arbel G., Hammerman H. (2011). Modelling the long QT syndrome with induced pluripotent stem cells. Nature.

[96] Zhang J., Lian Q., Zhu G., Zhou F., Sui L., Tan C., Mutalif R.A., Navasankari R., Zhang Y., Tse H.F. (2011). A human iPSC model of hutchinson gilford progeria reveals vascular smooth muscle and mesenchymal stem cell defects. Cell Stem Cell.

[97] Yamanaka S. (2009). A fresh look at ips cells. Cell.

[98] Yakhnenko I., Wong W.K., Katkov I.I., Itkin-Ansari P. (2012). Cryopreservation of human insulin expressing cells macro-encapsulated in a durable therapeutic immunoisolating device theracyte. CryoLetters.

[99] Nguyen H.N., Byers B., Cord B., Shcheglovitov A., Byrne J., Gujar P., Kee K., Schule B., Dolmetsch R.E., Langston W. (2011). Lrrk2 mutant iPSC-derived DA neurons demonstrate increased susceptibility to oxidative stress. Cell Stem Cell.

[100] Johannesson B., Sui L., Freytes D.O., Creusot R.J., Egli D. (2015). Toward β cell replacement for diabetes. Embo J..

[101] Finsterer J. (2009). Manifestations of the mitochondrial a3243g mutation. Int. J. Cardiol..

[102] Fujikura J., Nakao K., Sone M., Noguchi M., Mori E., Naito M., Taura D., Harada-Shiba M., Kishimoto I., Watanabe A. (2012). Induced pluripotent stem cells generated from diabetic patients with mitochondrial DNA a3243g mutation. Diabetologia.

[103] Hussein S.M., Batada N.N., Vuoristo S., Ching R.W., Autio R., Narva E., Ng S., Sourour M., Hamalainen R., Olsson C. (2011). Copy number variation and selection during reprogramming to pluripotency. Nature.

[104] Gore A., Li Z., Fung H.L., Young J.E., Agarwal S., Antosiewicz-Bourget J., Canto I., Giorgetti A., Israel M.A., Kiskinis E. (2011). Somatic coding mutations in human induced pluripotent stem cells. Nature.

[105] Lister R., Pelizzola M., Kida Y.S., Hawkins R.D., Nery J.R., Hon G., Antosiewicz-Bourget J., O'Malley R., Castanon R., Klugman S. (2011). Hotspots of aberrant epigenomic reprogramming in human induced pluripotent stem cells. Nature.

[106] Pera M.F. (2011). Stem cells: The dark side of induced pluripotency. Nature.

[107] Chun Y.S., Chaudhari P., Jang Y.Y. (2010). Applications of patient-specific induced pluripotent stem cells; focused on disease modeling, drug screening and therapeutic potentials for liver disease. Int. J. Biol. Sci..

[108] Abdelalim E.M., Bonnefond A., Bennaceur-Griscelli A., Froguel P. (2014). Pluripotent stem cells as a potential tool for disease modelling and cell therapy in diabetes. Stem Cell Rev. Rep..

[109] Basford C.L., Prentice K.J., Hardy A.B., Sarangi F., Micallef S.J., Li X., Guo Q., Elefanty A.G., Stanley E.G., Keller G. (2012). The functional and molecular characterisation of human embryonic stem cell-derived insulin-positive cells compared with adult pancreatic beta cells. Diabetologia.

[110] Marquez-Aguirre A.L., Canales-Aguirre A.A., Padilla-Camberos E., Esquivel-Solis H., Diaz-Martinez N.E. (2015). Development of the endocrine pancreas and novel strategies for β-cell mass restoration and diabetes therapy. Braz. J. Med. Biol. Res..

[111] Pagliuca F.W., Melton D.A. (2013). How to make a functional β-cell. Development.

[112] Weir G.C., Cavelti-Weder C., Bonner-Weir S. (2011). Stem cell approaches for diabetes: Towards β cell replacement. Genome Med..

[113] Thatava T., Kudva Y.C., Edukulla R., Squillace K., De Lamo J.G., Khan Y.K., Sakuma T., Ohmine S., Terzic A., Ikeda Y. (2013). Intrapatient variations in type 1 diabetes-specific iPS cell differentiation into insulin-producing cells. Mol. Ther..

[114] Wen Y., Chen B., Ildstad S.T. (2011). Stem cell-based strategies for the treatment of type 1 diabetes mellitus. Expert Opin. Biol. Ther..

[115] Liu X., Wang Y., Li Y., Pei X. (2013). Research status and prospect of stem cells in the treatment of diabetes mellitus. Sci. China-Life Sci..

[116] Goodrich A.D., Ersek A., Varain N.M., Groza D., Cenariu M., Thain D.S., Almeida-Porada G., Porada C.D., Zanjani E.D. (2010). In vivo generation of β-cell-like cells from CD34(+) cells differentiated from human embryonic stem cells. Exp. Hematol..

